# Subcortical Volumes Differ in Parkinson’s Disease Motor Subtypes: New Insights into the Pathophysiology of Disparate Symptoms

**DOI:** 10.3389/fnhum.2016.00356

**Published:** 2016-07-11

**Authors:** Keren Rosenberg-Katz, Talia Herman, Yael Jacob, Efrat Kliper, Nir Giladi, Jeffery M. Hausdorff

**Affiliations:** ^1^Center for the Study of Movement, Cognition and Mobility, Neurological Institute, Tel Aviv Sourasky Medical CenterTel Aviv, Israel; ^2^Functional Brain Center, Wohl Institute for Advanced Imaging, Tel Aviv Sourasky Medical CenterTel Aviv, Israel; ^3^Sagol School of Neuroscience, Tel Aviv UniversityTel Aviv, Israel; ^4^Neurological Institute, Tel Aviv Medical CenterTel Aviv, Israel; ^5^Department of Neurology, Sackler Faculty of Medicine, Tel Aviv UniversityTel Aviv, Israel; ^6^Department of Physical Therapy, Sackler Faculty of Medicine, Tel Aviv UniversityTel Aviv, Israel

**Keywords:** Parkinson’s disease, tremor, postural instability gait difficulty, volumetric MRI, basal ganglia, globus pallidus, imaging, gait

## Abstract

**Objectives**: Patients with Parkinson’s disease (PD) can be classified, based on their motor symptoms into the Postural Instability Gait Difficulty (PIGD) subtype or the Tremor Dominant (TD) subtype. Gray matter changes between the subtypes have been reported using whole brain Voxel-Based Morphometry (VBM), however, the evaluation of subcortical gray matter volumetric differences between these subtypes using automated volumetric analysis has only been studied in relatively small sample sizes and needs further study to confirm that the negative findings were not due to the sample size. Therefore, we aimed to evaluate volumetric changes in subcortical regions and their association with PD motor subtypes.

**Methods**: Automated volumetric magnetic resonance imaging (MRI) analysis quantified the subcortical gray matter volumes of patients with PD in the PIGD subtype (*n* = 30), in the TD subtype (*n* = 30), and in 28 healthy controls (HCs).

**Results**: Significantly lower amygdala and globus pallidus gray matter volume was detected in the PIGD, as compared to the TD subtype, with a trend for an association between globus pallidus degeneration and higher (worse) PIGD scores. Furthermore, among all the patients with PD, higher hippocampal volumes were correlated with a higher (better) dual tasking gait speed (*r* = 0.30, *p* < 0.002) and with a higher global cognitive score (*r* = 0.36, *p* < 0.0001). Lower putamen volume was correlated with a higher (worse) freezing of gait score (*r* = −0.28, *p* < 0.004), an episodic symptom which is common among the PIGD subtype. As expected, differences detected between HCs and patients in the PD subgroups included regions within the amygdala and the dorsal striatum but not the ventral striatum, a brain region that is generally considered to be more preserved in PD.

**Conclusions**: The disparate patterns of subcortical degeneration can explain some of the differences in symptoms between the PD subtypes such as gait disturbances and cognitive functions. These findings may, in the future, help to inform a personalized therapeutic approach.

## Introduction

Patients with Parkinson’s disease (PD) can be classified based on their motor symptoms into the Tremor Dominant (TD) or the Postural Instability Gait Difficulty (PIGD) subtype, depending on whether tremor or balance and gait disturbances are the most pronounced symptoms (Jankovic et al., 1990). Patients with the PIGD subtype have an increased risk for developing cognitive deterioration (Lewis et al., 2005; Burn et al., 2006, 2012; Herman et al., 2015), dementia (Aarsland et al., 2003, 2009; Williams-Gray et al., 2007) and mood disturbances such as depression or anxiety (Burn et al., 2012). Thus, in addition to the differences in PD motor symptoms, behavioral evidence suggests that the neurological substrate differs in the PIGD and TD subtypes.

These motor, cognitive, and behavioral differences among the PD subtypes likely reflect alternations in brain structure. Structural changes detected using Magnetic Resonance Imaging (MRI) have been used as biological markers of neurodegeneration in PD (Whitwell and Josephs, 2007; Pereira et al., 2012). Using Voxel-Based Morphometry (VBM), we recently reported on widespread gray matter reduction in the PIGD subtype, as compared to the TD subtype, in the frontal, parietal, occipital, and temporal lobes as well as in the parahippocampal gyrus, cerebellum, caudate nucleus and amygdala (Rosenberg-Katz et al., 2013). While VBM enables us to evaluate whole brain changes, it may suffer from errors that might arise from registration and segmentation procedures, especially in subcortical regions. Automated volumetric analysis is considered to be less sensitive to registration errors and to anatomical variability such as ventricular enlargement which is common in neurological diseases and aging (Khan et al., 2008). Therefore, while this technique also has its limitations, it has been considered to be the gold-standard approach for morphological analysis of MRI data in older adults (Fischl et al., 2002, 2004). Recently, a study using this automated approach found no differences in subcortical brain volumes between the PD subtypes (Nyberg et al., 2015), however, the relatively small number of subjects (12 and 9 for the PIGD and TD groups, respectively) might explain the negative findings. Additional study, using a larger cohort of patients with PD, is needed in order to determine if the previously reported absence of subcortical brain volume differences between the subtypes was due to a small sample size.

In the current study, we used automated volumetric analysis to evaluate differences in subcortical degeneration between the TD (*n* = 30) and PIGD (*n* = 30) subtypes. Based on our previous work, we expected to find a reduction in subcortical gray matter volume in the PIGD compared to the TD subtype. Specifically, we hypothesized that the PIGD subtype will have smaller gray matter volumes within the caudate nucleus (an area which is related to cognitive function), within the amygdala (an area which may be involved in the affective symptoms that are more common in this group), and within the globous pallidus (a brain area that might relate to the gait disturbances of the PIGD subtype as part of its role in the sensorimotor and the associative circuit; Tremblay et al., 2015).

## Materials and Methods

One-hundred and ten patients with idiopathic PD and 28 healthy controls (HCs) were recruited for this study. This is a secondary analysis of work designed to compare PD motor subtypes (Herman et al., 2013; Rosenberg-Katz et al., 2013). All patients were diagnosed by a movement disorders specialist as having idiopathic PD (as defined by the UK Brain Bank criteria). Patients and controls were excluded if they had major orthopedic disease, acute illness, history of stroke, a diagnosis of dementia based on DSM-IV criteria or a Mini Mental State Examination score (MMSE) < 24 (Folstein et al., 1983), had a diagnosed psychiatric disorder, or if they underwent brain surgery in the past. For the HCs, exclusion criteria also included any neurological disease.

### Protocol Outline

All subjects provided informed written consent prior to participating in the study, as approved by the Human Research Ethics Committee of Tel Aviv Sourasky Medical Center. Patients were studied on two separate occasions: the first visit included a neurological and clinical examination. On a separate visit that took place within 2 weeks after the first visit, the participants underwent MRI testing in the “ON” medication state.

### Clinical Evaluation

Patients underwent a clinical assessment that included the Unified Parkinson’s Disease Rating Scale (UPDRS). The pull test (item 30 of the UPDRS) was used as a measure of balance and postural control. Usual and dual tasking gait speed (m/s) under single and dual task conditions (i.e., serial 3 subtracting) was determined as a measure of gait difficulties (Herman et al., 2014). These assessments were conducted in the “OFF” state after at least 12 h of overnight withdrawal of anti-parkinsonian medications. A computerized cognitive battery (NeuroTrax Corp., Modiin, Israel) (Dwolatzky et al., 2003) was used in the “ON” state to sample a wide range of cognitive domains and to generate a normalized global cognitive score. To evaluate confidence while walking and fear of falling, the Activities-specific Balance Confidence (ABC) scale (Powell and Myers, 1995) was used. Emotional well-being and depressive symptoms were assessed using the 15-item version of the Geriatric Depression Scale (Yesavage et al., 1982). Balance and postural control were evaluated using the Berg Balance Scale (BBS; Berg et al., 1992). Patients also completed the New Freezing of Gait Questionnaire (N-FOGQ; Nieuwboer et al., 2009). The daily levodopa equivalent dosage (LED) was calculated for each patient as previously described (Tomlinson et al., 2010).

### Classification into PIGD and TD Subtypes

From the 110 patients that were recruited for this study, automated volumetric analysis was conducted on 105 patients (5 patients did not complete the MRI scans). These patients were classified into the PIGD or TD subtypes as previously described (Rosenberg-Katz et al., 2013). Briefly, symptoms were first quantified by summing specific items from the UPDRS to determine PIGD and TD scores that reflect gait and balance difficulties and tremor severity, respectively (Jankovic et al., 1990). Patients were classified to PIGD or TD group based on the ratio between the PIGD and tremor scores. Then, in order to stratify the patients into groups who best represented the two subtypes, with minimal overlap across symptom classes, patients were excluded from the TD group if they had a PIGD score higher than 3 or a tremor score lower than 4. Similarly, patients were excluded from the PIGD group if their PIGD score was lower than 4 or their tremor score was higher than 3 (Rosenberg-Katz et al., 2013). Patients who did not meet the criteria for one of the groups were considered as indeterminate. Thirty PIGD patients, 30 TD patients, and 45 indeterminate patients were identified.

### MRI Acquisition

All of the MR images were acquired on a 3.0 T (GE) scanner using an 8-channel head coil. A high-resolution T1-weighted brain volume (BRAVO) acquisition was used with the following parameters: repetition time (TR) = 9000 ms, echo time (TE) = 3.6 ms, flip angle (FA) = 90°, voxel size = 1 × 1 × 1, matrix = 256 × 256, field of view (FOV) = 250 × 250 mm^2^.

### Volumetric Analysis

The automated volume-based stream was performed on axial 3D T1-weighted BRAVO images using the FreeSurfer V5.1 image analysis suite, well documented and freely available software^1^ (Fischl et al., 2002, 2004).

Processing included affine transformation of each participant’s T1 weighted image into Talairach space, probabilistic segmentation of gray and white matter structures, bias field intensity normalization, and automated labeling of anatomical regions of interest (ROIs) in both cerebral hemispheres (Fischl et al., 2002, 2004). The ROIs derived from this analysis were the same as those examined by Nyberg et al. (2015) and included the thalamus, the caudate nucleus, the putamen, the globus pallidus, the amygdala, the nucleus accumbens, and the hippocampus. While there is some disagreement about whether the hippocampus should be considered as a subcortical region, it was included as it is part of the limbic system and to compare with the work of Nyberg et al. (2015). For each patient, the ROIs contralateral to the more affected side, defined by the side with a higher score on the UPDRS, were examined. For the HCs, and patients with no dominant affected side, the mean value of both hemispheres was used for comparisons.

### Statistical Analysis

All statistical analyses were two-sided and conducted using the Statistical Package for Social Sciences (Version 20; SPSS Inc., Chicago, IL, USA). Multivariate analysis of covariance (MANCOVA) adjusted for age and total intracranial volume was used to compare ROIs volumes across PIGD, TD subgroups and controls. Similar analyses which also included the indeterminate patients were also performed to evaluate if this group had ROIs volumes in between the values seen in the two subtypes. As these regions were predefined, no correction for multiple comparisons was performed in this analysis. For all correlation analyses, partial correlations were conducted adjusting for age and total intracranial volume. For patients with PD, correlations between gray matter brain volumes and PIGD score, tremor score, gait speed (usual and dual tasking), N-FOGQ, global cognitive score, ABC scale, and Geriatric Depression Scale (GDS) were conducted in the entire sample (*n* = 105), including the indeterminate group, and were adjusted for age, disease duration and total intracranial volume. Outliers were defined as values larger than 2 interquartile ranges above the 75th percentile or lower than 2 interquartile ranges below the 25th percentile, however, no outliers were detected. Bonferroni corrections for multiple comparisons were also applied for these correlation analyses.

## Results

### Patient Characteristics

There were no significant differences between the PIGD and TD groups in their demographic characteristics including age, gender, years of education, UPDRS motor scores, disease duration, and LED (see Table 1). As expected, axial motor impairments were more severe in the PIGD group than in the TD group, as measured by the pull test and the BBS. Usual and dual tasking gait speeds were lower in the PIGD subtype. The executive function index from the computerized cognitive battery, Mini Mental Status Exam (MMSE) scores and global cognitive scores tended to be lower in the PIGD group than in the TD group; however, these group differences were not significant (Table 1). Patients with the PIGD subtype also had worse scores on the ABC scale and GDS, respectively.

**Table 1:** **Demographic characteristics of the study participants**.

	Healthy controls	Indeterminate patients	PIGD	TD	*p*-value (PIGD vs. TD)
Number of subjects	28	45	30	30	
Age (years)	64.91 ± 8.76	65.16 ± 8.43	64.95 ± 7.71	64.60 ± 8.85	0.90
Female/male	12/16	36/9	12/18	7/23	0.08
Years of education	16.01 ± 3.45	15.34 ± 3.22	15.80 ± 3.90	15.00 ± 3.30	0.33
Disease duration (years)	NA	5.62 ± 5.50	5.69 ± 3.68	5.36 ± 3.15	0.90
LED (mg)	NA	343.00 ± 430.73	582.12 ± 341.45	488.23 ± 284.13	0.25
UPDRS motor sum	NA	41.85 ± 14.70	38.74 ± 10.47	39.47 ± 15.30	0.80
PIGD score	NA	4.65 ± 2.58	7.29 ± 3.10	1.84 ± 0.88	0.00001
Tremor score	NA	7.43 ± 5.97	1.52 ± 0.93	11.88 ± 3.55	0.00001
New freezing of gait questionnaire	NA	4.50 ± 7.66	8.16 ± 9.88	0 ± 0	0.0001
Usual-walking gait speed (m/s)	1.15 ± 0.14	1.09 ± 0.21	1.07 ± 0.19	1.21 ± 0.19	0.002
Dual tasking gait speed (m/s)	1.07 ± 0.19	0.98 ± 0.25	0.90 ± 0.27	1.06 ± 0.20	0.009
Global cognitive score	95.88 ± 9.43	94.49 ± 10.70	90.04 ± 14.39	95.89 ± 10.79	0.073
Geriatric depression scale	NA	3.96 ± 3.20	4.70 ± 3.14	3.00 ± 3.19	0.04
Activities-specific balance confidence scale	NA	84.50 ± 16.50	79.20 ± 17.00	94.55 ± 7.84	0.0003

*LED, Levodopa equivalent dosage; UPDRS, Unified Parkinson’s Disease Rating Scale; NA, not available*.

### Volumetric Analysis

**Figure 1:**
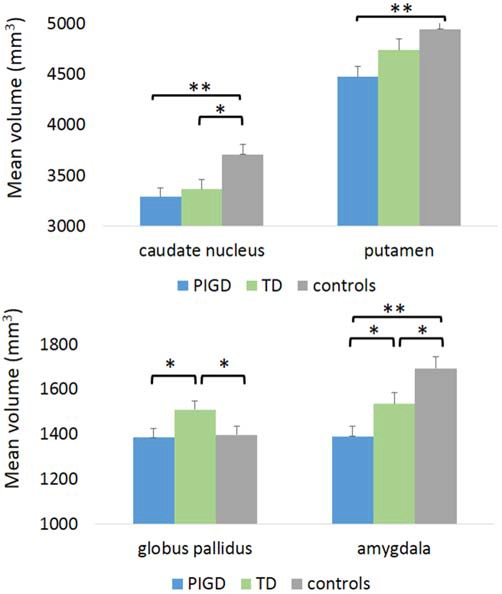
**Volumetric differences in selected regions between the TD subtype, the PIGD subtype and controls.** TD, tremor dominant; PIGD, Postural Instability Gait Difficulty. *p < 0.05, **p < 0.005.

MANCOVA analysis including the PIGD group, the TD group, and HCs detected a significant GROUP effect within the caudate, putamen, globus pallidus and amygdala. The estimated marginal means adjusted for age and intracranial volume are presented in Table 2 and Figure 1. *Post hoc* analysis showed that the PIGD patients had lower gray matter volumes in the globus pallidus and amygdala, as compared to the patients with the TD subtype. Both the PIGD and TD subtypes had lower caudate nucleus and amygdala volumes, as compared to HCs. The PIGD subtype also had a lower putamen gray matter volume compared to the controls (see Table 3 and Figure 1).

**Table 2:** **Volumetric comparisons in the selected regions of interest (ROIs) between the postural instability gait difficulty (PIGD) subtype, tremor dominant (TD) subtype and HCs**.

Region	Estimated mean controls (*n* = 28)	Estimated mean PIGD (*n* = 30)	Estimated mean TD (*n* = 30)	Group (healthy controls, PIGD and TD subtypes) *F* value (*p*-value)
Cerebellum	50513 ± 1420	49304 ± 1317	49567 ± 1403	0.21 (0.81)
Thalamus	6303 ± 126	6430 ± 118	6653 ± 89	1.78 (0.18)
Caudate	3707 ± 99	3287 ± 91	3363 ± 97	**5.34 (0.007)**
Putamen	4947 ± 109	4475 ± 101	4738 ± 108	**5.29 (0.007)**
Globus pallidus	1397 ± 37	1387 ± 35	1509 ± 37	**3.20 (0.046)**
Hippocampus	3824 ± 52	3724 ± 87	3817 ± 77	0.41 (0.66)
Amygdala	1693 ± 50	1391 ± 46	1535 ± 50	**9.95 (0.0001)**
Nucleus accumbens	586 ± 20	539 ± 18	561 ± 19	1.63 (0.20)

**Table 3:** ***p*-values for *post hoc* comparisons between the PIGD subtype, TD subtype and HCs**.

Region	PIGD vs. TD	PIGD vs. controls	TD vs. controls
Cerebellum	n.s	n.s	n.s
Thalamus	n.s	n.s	n.s
Caudate	n.s	0.001	0.020
Putamen	n.s (*p* = 0.08)	0.002	n.s
Globus pallidus	0.02	n.s	0.045
Hippocampus	n.s	n.s	n.s
Amygdala	0.04	0.0003	0.036
Nucleus accumbens	n.s	n.s	n.s

*n.s, non-significant*.

**Figure 2:**
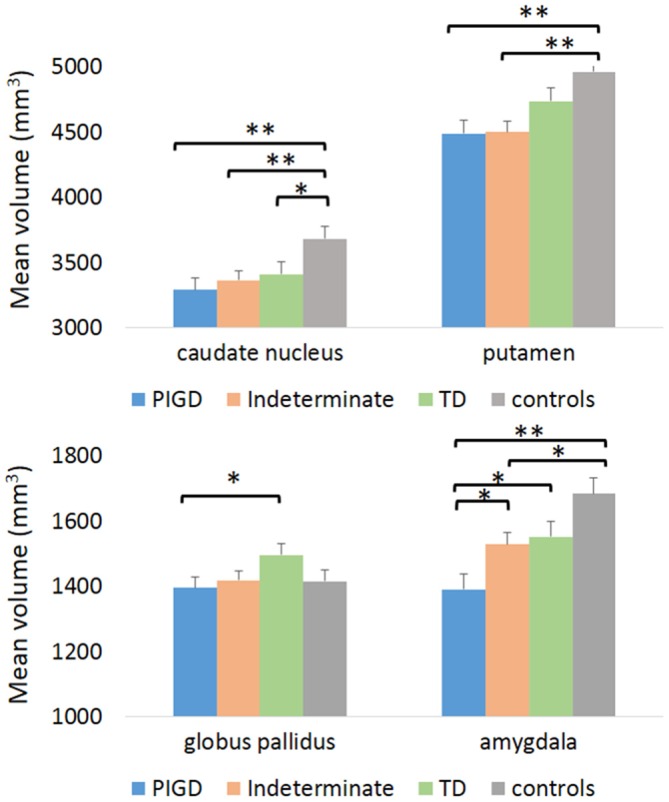
**Volumetric differences in selected regions between the TD subtype, the PIGD subtype, indeterminate patients and controls.** TD, tremor dominant; PIGD, Postural Instability Gait Difficulty. *p < 0.05, **p < 0.005.

Additional MANCOVA analyses which included the indeterminate group as well as the PIGD subtype, the TD subtype, and HCs showed a similar pattern of results with a significant GROUP effect within the caudate, putamen, and amygdala (see Tables 4, 5 and Figure 2). While the globus pallidus only showed a small trend for a group effect (*p* = 0.19), *post hoc* comparisons still showed a significant difference between the PIGD and TD subtypes (*p* < 0.04, see Table 5) within this region. Interestingly, in the regions showing a significant GROUP effect, the volumes of the indeterminate group were between the volumes of the two subtypes (see Table 4 and Figure 2). Furthermore, the amygdala volume was significantly larger in the indeterminate group than in the PIGD subtype, while no significant difference between the indeterminate group and the two PD subtypes were detected for the other regions (see Table 5).

**Table 4:** **Volumetric comparisons in the selected ROIs between the PIGD subtype, TD subtype, indeterminate patients and HCs**.

Region	Estimated Mean controls (*n* = 28)	Estimated Mean PIGD (*n* = 30)	Estimated Mean indeterminate (*n* = 45)	Estimated Mean TD (*n* = 30)	Group (healthy controls, PIGD, indeterminate, and TD subtypes) *F* value (*p*-value)
Cerebellum	50768 ± 1556	49444 ± 1457	50709 ± 1186	49617 ± 1512	0.25 (0.86)
Thalamus	6330 ± 129	6447 ± 121	6376 ± 99	6647 ± 126	1.24 (0.29)
Caudate	3682 ± 95	3290 ± 89	3364 ± 72	3413 ± 92	**3.50 (0.02)**
Putamen	4960 ± 109	4487 ± 102	4498 ± 83	4735 ± 106	**5.05 (0.002)**
Globus pallidus	1415 ± 35	1396 ± 33	1419 ± 27	1496 ± 34	1.64 (0.19)
Hippocampus	3808 ± 65	3722 ± 80	3907 ± 65	3841 ± 83	1.09 (0.36)
Amygdala	1684 ± 49	1391 ± 46	1529 ± 37	1551 ± 48	**6.63 (0.0003)**
Nucleus accumbens	589 ± 20	540 ± 19	556 ± 15	560 ± 20	1.13 (0.34)

**Table 5:** ***p*-values for *post hoc* comparisons between the PIGD subtype, TD subtype, indeterminate patients and HCs**.

Region	PIGD vs. TD	PIGD vs. controls	TD vs. controls	Indeterminate vs. controls	PIGD vs. indeterminate	TD vs. indeterminate
Cerebellum	n.s	n.s	n.s	n.s	n.s	n.s
Thalamus	n.s	n.s	n.s	n.s	n.s	n.s
Caudate	n.s	0.003	0.05	0.009	n.s	n.s
Putamen	n.s (*p* = 0.2)	0.002	n.s	0.001	n.s	n.s
Globus pallidus	0.04	n.s	n.s	n.s	n.s	n.s
Hippocampus	n.s	n.s	n.s	n.s	n.s	n.s
Amygdala	0.018	0.0002	n.s (0.059)	0.013	0.02	n.s
Nucleus accumbens	n.s	n.s	n.s	n.s	n.s	n.s

**Figure 3:**
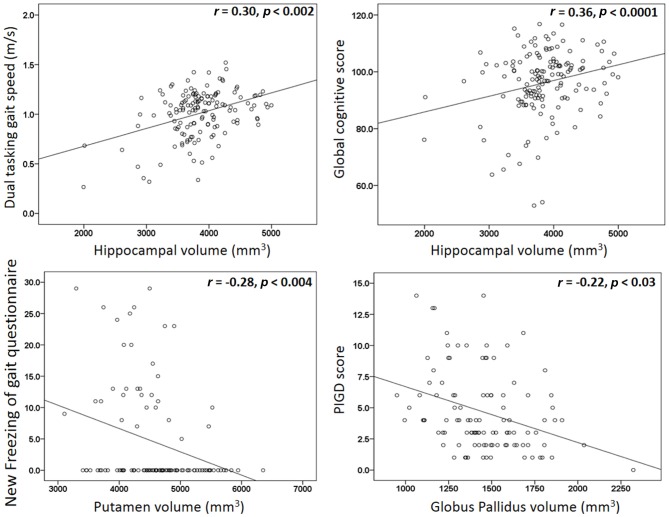
**Scatter plots for the significant correlations detected between gray matter volumes and behavioral measurements**.

Among all of the patients with PD (*n* = 105), higher hippocampal volumes were correlated with higher dual tasking gait speed (*r* = 0.30, *p* < 0.002) and with higher global cognitive score (*r* = 0.36, *p* < 0.0001; see Figure 3). Higher (worse) scores on the N-FOGQ were correlated with lower putamen gray matter volumes (*r* = −0.28, *p* < 0.004; see Figure 3). No significant correlations were detected between gray matter volumes and tremor or the PIGD scores, however, using a more liberal threshold, the PIGD score was mildly associated with lower globus pallidus volume (*r* = −0.22, *p* < 0.03; see Figure 3), but not when corrected for multiple comparisons.

## Discussion

In this study, greater amygdala and globus pallidus gray matter loss were detected in the PIGD, as compared to the TD subtype. In addition, the PIGD subtype had significantly higher putamen degeneration than controls. Interestingly, the putamen volumes in the TD subtype were not significantly different than controls. Furthermore, increased putamen degeneration was associated with a higher (worse) freezing of gait score, an episodic symptom which is more associated with the PIGD subtype and likely involves a motor-cognitive failure (Giladi et al., 2007; Fasano et al., 2011; Cohen et al., 2014; Maidan et al., 2015). As might be expected, indeterminate PD patients, who have a mixture of symptoms of both subtypes, had an intermediate volume between the subtypes in regions where a significant difference between the groups was observed.

Patients with PD had lower subcortical volumes within the amygdala and the dorsal striatum (caudate and putamen), as compared to HCs. The dorsal striatum is believed to be especially sensitive to PD, and this atrophy is considered to be a marker for neurodegeneration, as it is shown to be associated with the stages and severity of the disease (Pereira et al., 2012). In contrast, no differences were found within the ventral striatum (nucleus accumbens) which is considered to be more preserved in PD, based on the localized accumulation of Lewy bodies (Braak et al., 2003). Based on the role of the caudate nucleus in cognition as part of the associative corticostriatal circuit (Alexander et al., 1986; Tremblay et al., 2015), we would expect to find reduced caudate nucleus volumes in patients of the PIGD subtype, as they have a higher risk for developing cognitive impairment (Lewis et al., 2005; Burn et al., 2006, 2012; Herman et al., 2015). Perhaps the role of the caudate nucleus in cognition is also affected by its functional connectivity with other brain regions, as previously demonstrated in patients with PD (Vervoort et al., 2016).

The increased pallidal degeneration in the PIGD subgroup, as compared to the TD subgroup, may partially explain a range of motor and non-motor symptoms common in the PIGD subgroup. Indeed, the globus pallidus is involved in the sensorimotor, associative and limbic corticostriatal circuits (Alexander et al., 1986). These three circuits play a role in many aspects of action planning, starting from motivational information for goal selection through the limbic circuit, continuing with action selection via the associative circuit, and concluding with movement selection and execution by the sensorimotor circuit (Tremblay et al., 2015). The gait disturbances, which are highly representative of the PIGD subtype, can be related to both the impairment of the sensorimotor and the associative circuit.

Amygdala degeneration was detected in both subtypes, as compared to HCs, with greater degeneration in the PIGD subtype, compared to the TD subtype and to the indeterminate group. This is consistent with a previous report that compared PD subjects without dementia to controls (Bouchard et al., 2008) and with our previous VBM analysis (Rosenberg-Katz et al., 2013). The detected changes in amygdala volume can be related to a number of affective (non-motor) symptoms in PD, including depression, apathy, compulsive behavior, and anxiety (Chaudhuri et al., 2006). Depression and anxiety were more severe in the PIGD subtype than in the TD subtype (Burn et al., 2012), which is consistent with our findings of greater level of fear of falling (reflected by the lower balance confidence scores) and higher depression scores in the PIGD subtype. Nonetheless, we could not detect any significant correlations between these measurements and amygdala gray matter volume. Perhaps a latent variable, possibly within the limbic system, mediates those relationships.

The detected correlations between hippocampal volumes with both global cognitive score and dual tasking gait speed are consistent with previously reported associations between lower hippocampus volume and reduced gait speed and step length in non-PD older adults (Callisaya et al., 2013). Similarly, lower hippocampal volume and metabolism (measured using MR spectroscopy) have been associated with poorer stride length in older adults without dementia (Zimmerman et al., 2009). Overall, our findings support the suggested relationships between gait and cognition (Amboni et al., 2013), possibly via the role of the hippocampus in spatial orientation and memory (O’Keefe et al., 1998). The lack of hippocampal differences between the groups is consistent with other volumetric studies in PD, which found lower hippocampus volumes in patients with PD-related dementia as compared to patients with no dementia (Zarei et al., 2013; Xu et al., 2016), while no changes were detected when comparing PD patients with mild cognitive impairment to PD patients with no cognitive impairment, suggesting that the hippocampal atrophy in PD is a gradual progressive process (Xu et al., 2016).

Interestingly, higher globus pallidus volumes were detected in the TD group, as compared to both controls and patients in the PIGD group. This observation is consistent with previous VBM studies which showed that PD tremor and essential tremor are associated not only with atrophy of brain regions but also with cortical enlargement. For example, enlargement of posterior parts of the thalamus was present in PD patients with tremor, as compared to controls (Kassubek et al., 2002; Lin et al., 2013). In our cohort, there was a trend for increased thalamic volume in the tremor subtype, although this was not significant. The direct relationship between the globus pallidus and the thalamus within the direct basal ganglia-thalamocortical circuit may explain the enlargement of both of these regions. This enlargement might reflect a compensatory mechanism in response to damage to the basal ganglia-thalamocortical circuit. Another possibility is that patients in the TD subtype are initially protected from globus pallidus and thalamic degeneration and this is why they have early tremor and not PIGD symptoms. Indeed, a functional MRI study reported higher functional activation of the globus pallidus in patients with tremor dominant PD, as compared to those without (Prodoehl et al., 2013). The nature of this enlargement, either as a result of neuronal hypertrophy or by a higher density of neurons, can only be determined using post-mortem pathological investigations.

The volumetric differences that were found in the present study between the PD subtypes are not in agreement with a recent study which did not find volumetric changes in these same subcortical regions (Nyberg et al., 2015). Several possibilities might explain this discrepancy. First, our analysis included 30 patients in each group of the PD subtypes while Nyberg et al. (2015) included only nine PIGD and 12 TD patients. Second, we used additional restrictions for the traditional stratification into the PD subtypes (Jankovic et al., 1990), in order to exclude patients with mixed symptoms from the analysis (Rosenberg-Katz et al., 2013). This stricter stratification may enable the detection of subtle changes between the two PD subtypes. Further work is needed to confirm the results of the present study and better understand these differences.

Previous whole-brain VBM analysis on the same cohort (Rosenberg-Katz et al., 2013) detected widespread frontal, parietal, occipital, and temporal gray matter reduction in the PIGD subtype as compared to the TD subtype, as well as changes in the parahippocampal gyrus, cerebellum, caudate nucleus, and amygdala. While the changes in the amygdala were consistent using both types of analysis, we could not detect any group differences within the cerebellum and the caudate nucleus. This discrepancy could be related to the type of analysis that was used. Indeed, it has been suggested that automated volumetric analysis is less affected by anatomical variability, especially in subcortical structures (Fischl et al., 2002, 2004) than VBM.

To conclude, the detected gray matter atrophy in subcortical areas can potentially explain some of the motor, cognitive and affective symptoms that are different among the PD subtypes. These anatomical changes suggest that subcortical degeneration is not distributed similarly across the two PD motor subtypes and support the possibility that different therapeutic approaches should be considered for each of the motor PD subtypes.

## Author Contributions

KR-K, TH, JMH and NG designed the work; and KR-K, YJ, TH and EK analyzed the data. Participants were recruited and images were acquired by KR-K, YJ and TH. KR-K wrote the first draft. All authors revised the final version critically for important intellectual content. All authors approved the final submitted version, and agree to be accountable for its content.

## Funding

This study was supported by the Michael J. Fox Foundation for Parkinson’s Research.

## Conflict of Interest Statement

The authors declare that the research was conducted in the absence of any commercial or financial relationships that could be construed as a potential conflict of interest.

## Footnotes

^1^http://surfer.nmr.mgh.harvard.edu/
